# Diagnostic value of CT and MRI combined with serum LDH, NSE, CEA, and MYCN in pediatric neuroblastoma

**DOI:** 10.1186/s12957-023-03131-5

**Published:** 2023-08-18

**Authors:** Jumei Hao, Jing Sang, Xiajuan Xu, Aihua Bao

**Affiliations:** 1 Department of Pediatrics, Yantaishan Hospital, Yantai, 264003 China; 2 Department of ICU, Affiliated Qingdao Central Hospital of Qingdao University, Qingdao Cancer Hospital, Qingdao, 266000 China; 3 Department of Tumor Comprehensive Treatment (I), Affiliated Qingdao Central Hospital of Qingdao University, Qingdao Cancer Hospital, Qingdao, 266000 China; 4 Medical Imaging Department, Qingdao Women and Children’s Hospital, No 6 Tongfu Road, Qingdao, 266021 China

**Keywords:** Neuroblastoma, Pathological examination, CT, MRI, MYCN

## Abstract

**Background:**

To analyze the diagnostic value of computed tomography (CT), magnetic resonance imaging (MRI) combined with serum lactate dehydrogenase (LDH), neuron-specific enolase (NSE), carcinoembryonic antigen (CEA), and N-myc (MYCN) in the diagnosis of pediatric neuroblastoma.

**Methods:**

Fifty-two children diagnosed with neuroblastoma were selected as the neuroblastoma group. During the same period, 52 children who visited our hospital with abdominal distension, diarrhea, constipation, and vomiting but were finally excluded from neuroblastoma were selected as the control group. CT and MRI were performed on all children.

**Results:**

Fifty-two cases of neuroblastoma of the central nervous system were confirmed by pathological examination. The levels of LDH, NSE, CEA, and MYCN in the neuroblastoma group were clearly higher than those in the control group (*P* < 0.05). The results of CT and MRI combined with serum LDH, NSE, CEA, and MYCN were false positive in 10 cases and false negative in 6 cases, which were consistent with the pathological results. The sensitivity of CT and MRI combined with serum LDH, NSE, CEA, and MYCN in the diagnosis of neuroblastoma was notably higher than that of the three alone (*P* < 0.05).

**Conclusion:**

The imaging findings of CT and MRI in children with central nervous system neuroblastoma were definitely characteristic. MRI had higher diagnostic value than CT. The diagnostic value of CT and MRI combined with serum LDH, NSE, CEA, and MYCN was improved to some extent.

## Introduction

Neuroblastoma is a malignant tumor of the sympathetic nervous system in children originating from tissues and organs. Its main clinical manifestations are headache, elevated intracranial pressure, and vomiting, and the most common symptom is painless abdominal mass [1]. Neuroblastoma accounts for 15% of all childhood cancer deaths worldwide [2]. The prognosis of children younger than 18 months at the time of diagnosis of neuroblastoma is better than that of children diagnosed later, and the growth of age is in turn related to the poor prognosis [3]. Therefore, early and accurate diagnosis of neuroblastoma can improve the prognosis of children [4–10].

At this stage, in clinical practice, we mainly rely on imaging examination to determine the location of the lesion, so as to carry out surgical treatment. At present, CT and magnetic resonance imaging (MRI) are the main effective methods for clinical diagnosis and examination of neuroblastoma, and the two methods have different diagnostic characteristics [11]. CT scanning is more convenient, requires less time, and has high sensitivity to intratumoral calcification, which can detect about 85% of intratumoral calcification. MRI diagnosis has high flexibility, can realize multi-directional imaging, and is convenient to make comparison of patients’ soft tissue structure, with high accuracy. Combining the advantages and disadvantages of the two methods in the diagnosis of neuroblastoma, in order to improve the accuracy of diagnosis and the success rate of surgical treatment, a comprehensive analysis of the two diagnostic methods was carried out at the same time, so as to develop a more perfect surgical treatment plan.

MYCN is a marker closely related to neuroblastoma proposed in recent years. Many research reports show that MYCN amplification is a biological indicator to measure the therapeutic effect and prognosis of neuroblastoma [12–14]. Smad7 is closely related to chemoresistance and epithelial-mesenchymal transformation of glioblastoma [15]. The aim of this study was to summarize the imaging characteristics of neuroblastoma by analyzing the CT and magnetic resonance imaging features and serum LDH, NSE, CEA, and MYCN levels of 52 children with neuroblastoma, and to explore the clinical diagnostic value of combined detection in pediatric neuroblastoma.

## Materials and methods

### General information

Fifty-two children with highly suspected neuroblastoma of the central nervous system who were examined in our hospital were selected as the study subjects, and all of them were examined with CT and MRI.

Inclusion criteria are as follows: (1) Children with neuroblastoma were diagnosed by physical examination, CT examination, and finally by surgery or puncture pathology, which met the expert consensus on the diagnosis and treatment of pediatric neuroblastoma compiled by the Pediatric Oncology Professional Committee of the Chinese Anti-Cancer Association; (2) the clinical data of the children were perfect and their consciousness was clear, which met the research needs; and (3) no radiotherapy, chemotherapy or surgical treatment was received before inclusion.

Exclusion criteria are as follows: (1) patients with diseases of the nervous system, blood system or immune system; (2) patients with other serious organ diseases or tumors; (3) patients with congenital heart disease or organ malformations; (4) patients with mental disorders and communication disorders; (5) patients with insufficient and incomplete clinical data; (6) patients who have not undergone pathological examination or whose pathological diagnosis is not clear; and (7) patients with secondary changes in the tumor site, such as ulcers, bleeding, and erosion. This study was approved by the Medical Ethics Association of Henan Children’s Hospital and met relevant standards. All the parents of enrolled children signed informed consent.

### Research methods

#### CT scan

All patients were diagnosed by CT examination (256-row detector CT (Revolution CT, GE Healthcare). The parameters were set as follows: layer thickness was 5 mm, and interlayer thickness was 3 mm. The scanning parameters were 300 mA current, 120 kV voltage, 0.579 pitch, 512 × 512 matrix, and 240 × 240 mm FOV. Continuous scanning was performed starting from the base of the patient’s skull.

#### MRI examination

All patients underwent MRI examination before operation (GE Architect, 3.0 T). Scanning parameters were set to the following: layer thickness was 5 mm, the layer distance was 1 mm, and the conventional diagnostic test axis was used in plain scan, T1WI (TR = 540 ms, TE = 24 ms), T2WI (TR = 4200 ms, TE = 100 ms), and FLAIRR (TR = 6000 ms, TE = 120 ms, TI = 2000 ms). A nonionic iodine contrast agent was injected intravenously into the patient, and the patient was diagnosed with a comprehensive scan. All patients underwent tumor resection after diagnosis and confirmation, and the resected pathological tissues were sent for examination using Fohr fixation. Finally, the results of pathological tissue examination were compared with the results of CT and MRI diagnostic examination.

#### LDH level detection

Serum LDH level was determined by lactic acid substrate rate method using an automated biochemical analyzer.

#### GEA and NSE detection

Fasting venous blood (2–3 mL) was taken from the children in the morning without anticoagulation. Without anticoagulation, serum was separated 1 h after hemagglutination (3000 r/min, centrifugal radius 150 mm, 5 min). Serum GEA and NSE levels were detected by radioimmunoassay or electrochemical spectrophotometry.

#### MYCN mRNA expression

Fasting blood was collected from all children and left at room temperature for 30–60 min. After centrifuging at 3000r/min for 10 min, the serum was extracted and stored for later use. MYCN mRNA level was detected by quantitative real-time PCR (qRT-PCR) instrument (ABI 7300, USA). According to the manufacturer’s instructions, the total RNA was extracted by RNA extraction kit, and was reversely transcribed into cDNA by reverse transcription kit. A reaction system with a volume of 20μL was configured, and the reaction conditions were set at 95℃ for 5 min, 94℃ for 30 s, 60℃ for 30 s, and 72℃ for 30 s and with 45 cycles. The relative expression levels of target genes were calculated by 2^−△△Ct^. The primers sequences of MYCN and reference GAPDH were shown in Table 1.

**Table 1:** Primer sequence

	Forward primer 5’-3’	Reverse primer 5’-3’
MYCN	GCAGACCAGAGCGGCCAGCC	CAGZACGACTTGTAGCGTAC
GAPDH	CAGGTTGACGAGCCAGCTC	CGTGCAACGGCTGTGCCTGC

#### Observation index

1. The results of histopathology were analyzed.
2. The specificity, sensitivity, accuracy, negative predictive value, and positive predictive value of CT and MRI in the diagnosis of neuroblastoma of the central nervous system were compared.
3. The imaging features of neuroblastoma of central nervous system were analyzed.

### Statistical analysis

Excel 2010 was used to establish the database, and SPSS 22.0 software was used for statistical analysis. Measurement data were expressed as mean ± standard deviation (mean ± SD), and independent sample *t* test was used to compare results which consistent with normal distribution. Count data were expressed as frequency and percentage and compared by chi-square test. *P* < 0.05 indicated that the difference was statistically significant.

## Results

### General situation of children in the two groups

A total of 52 children with high suspicion of neuroblastoma who were examined in our hospital were selected as the research objects, and all were examined with CT and MRI. There were 30 males and 22 females. The average age was 7.72 ± 1.06 years, ranging from 2 months to 15 years. During the same period, 52 children who were visited our hospital with abdominal distension, diarrhea, constipation, and vomiting but were finally excluded from neuroblastoma were selected as the control group. There were 29 males and 23 females. The average age was 7.82 ± 1.01 years, ranging from 5 months to 15 years.

### Imaging features

**Fig. 1:**
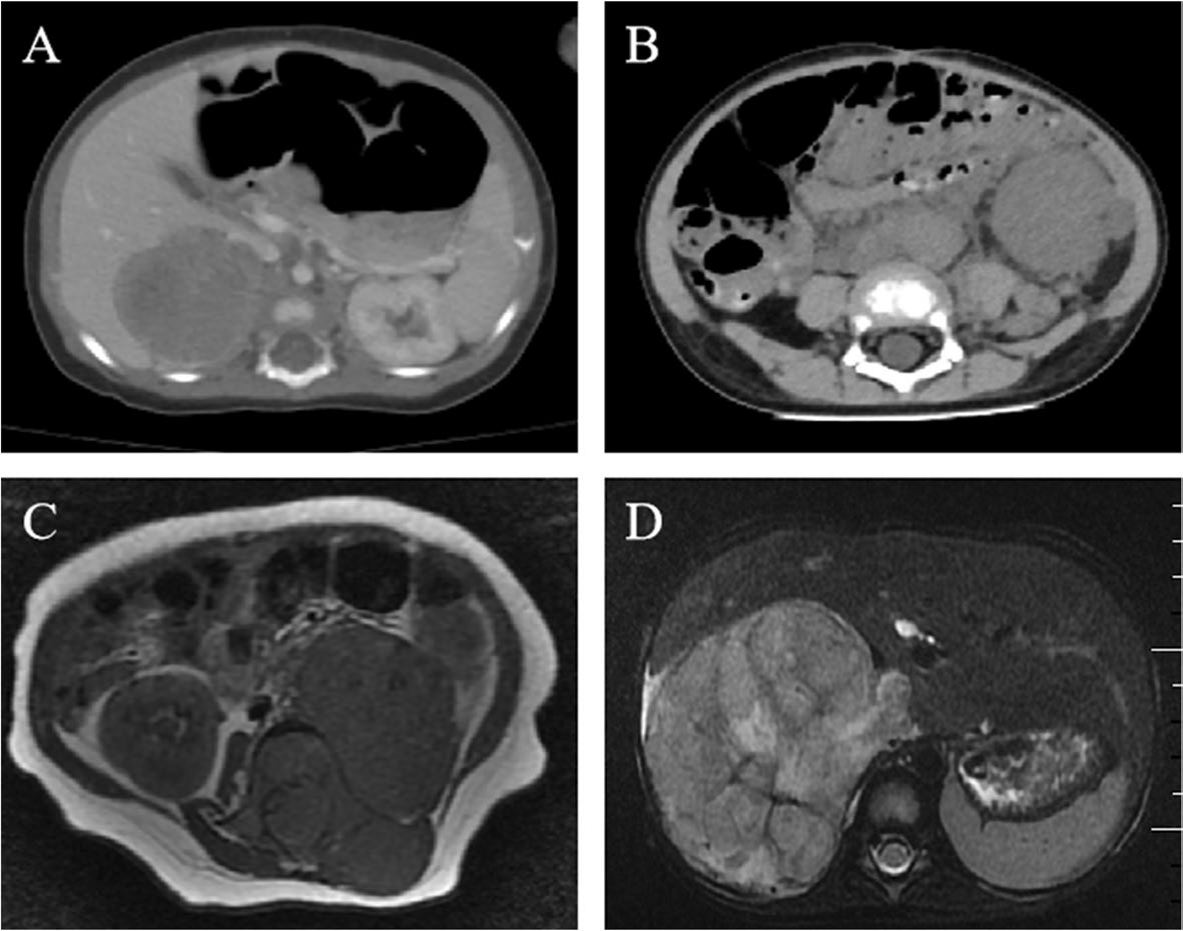
Imaging features. **A** CT image, male, 2 months, stage I, left adrenal neuroblastoma. The tumor density was not uniform, and irregular low-density areas were inside the tumor. **B** CT image, female, 15 months, stage III. An irregular mixed density mass with internal gritty calcification and marginal underfinish was observed retroperitoneally. **C** MRI image, female, 6 months, stage IV. A large substantial mass was behind the left peritoneum. **D** MRI image, female, 24 months, stage III, right adrenal neurocytoma. Plain scan TWI showed small lamellar vertebrae with slightly elevated signal transfer

CT examination of neuroblastoma of the central nervous system showed that the lesion was cystic and solid, the solid part was isodense, the cystic part was low density, the small strip of high-density calcification shadow was visible, and the edge of the pressed parietal bone was smooth. MRI examination showed the presence of lesions and swelling (4 cm × 3 cm × 3 cm) in the pontine. Scanning of the swelling showed that the lesion was mainly solid, with low signal on T1WI, high signal on T2WI, and isosignal on FLAIR. No enhanced lesion or surrounding tissue edema was observed on enhanced MRI scan (Fig. 1).

### Comparison of serum LDH, NSE and CEA levels between the two groups

**Fig. 2:**
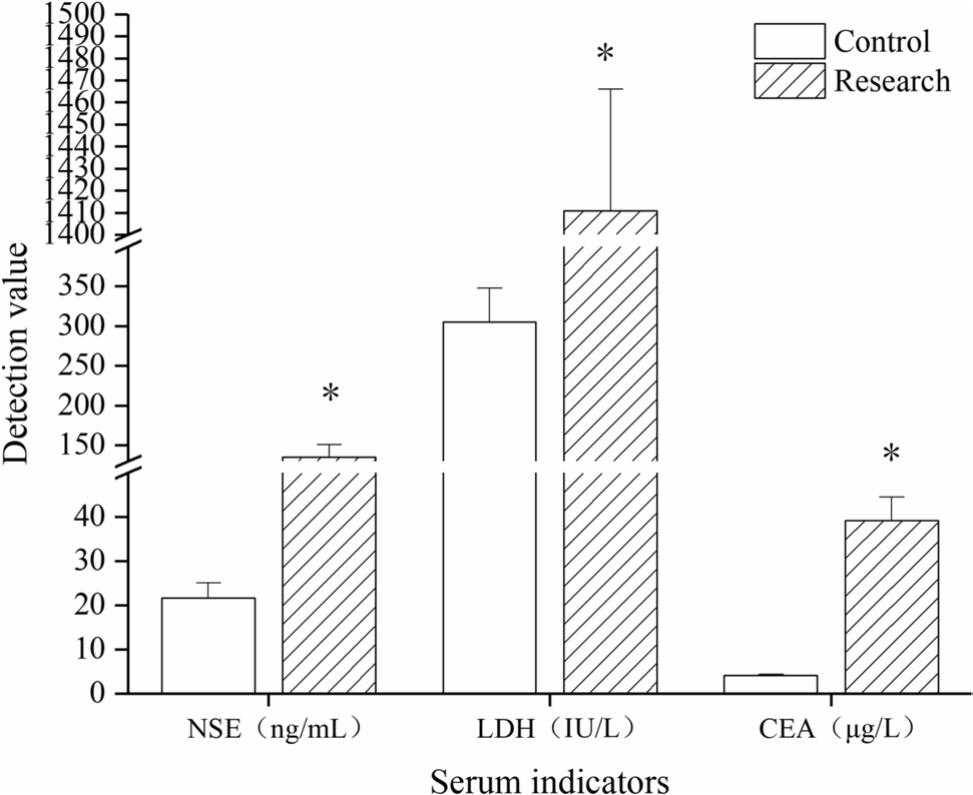
Comparison of serum LDH, NSE, and CEA levels between the two groups. Comparison of serum LDH, NSE, and CEA levels. *p < 0.05

LDH, NSE, and CEA3 indexes of 43 newly diagnosed neuroblastoma children were significantly higher than those of the normal control group (*P* < 0.05, Fig. 2). The positive rate of NSE was 67.2%, the positive rate of NSE + LDH combined test was 88.7%, and the positive rate of 3 combined tests was 92.5%, which was of great significance for clinical diagnosis.

### Diagnostic value of CT and MRI combined with serum MYCN mRNA in neuroblastoma

Serum MYCN mRNA level in the neuroblastoma group was significantly higher than that in the control group (*P* < 0.05). The results of CT combined with serum MYCN were false positive in 10 cases and false negative in 6 cases. The results of MRI combined with serum MYCN were false positive in 7 cases and false negative in 4 cases. The results of CT and MRI combined with serum MYCN were false positive in 4 cases and false negative in 2 cases (Table 2).

**Table 2:** Diagnostic value of CT and MRI combined with serum MYCN mRNA in neuroblastoma

Diagnostic method	Pathological results	
	Positive (*n* = 52)	Negative (*n* = 52)
CT + MYCN		
Positive	46	10
Negative	6	42
MRI + MYCN		
Positive	48	7
Negative	4	45
CT + MRI + MYCN		
Positive	50	4
Negative	2	48

### Comparison of efficacy of CT and MRI combined with serum LDH, NSE, CEA, MYCN, and mRNA alone and combined in the diagnosis of neuroblastoma

The sensitivity of CT and MRI combined with serum LDH, NSE, CEA, and MYCN mRNA in the diagnosis of neuroblastoma was significantly higher than that of CT and MRI combined (*P* < 0.05, Table 3).

**Table 3:** Comparison of efficacy of CT and MRI combined with serum LDH, NSE, CEA, and MYCN in the diagnosis of neuroblastoma

Diagnostic method	Specificity	Sensitivity	Accuracy	Positive predictive value	Negative predictive value
CT combined with serum markers	80.8 (42/52)	90.4 (47/52)	85.6 (89/104)	82.4 (47/57)	89.4 (42/47)
MRI combined with serum markers	90.4 (47/52)	92.3 (48/52)	91.3 (95/104)	90.6 (48/53)	92.2 (47/51)
CT and MRI combined serum markers	96.2 (50/52)	98.1 (51/52)	97.1 (101/104)	96.2 (51/53)	98.0 (50/51)

## Discussion

Although neuroblastoma has a low incidence, it is still the most common solid tumor in childhood, with a median age of onset of 23 months, and less than 10% of diagnosed cases after 5 years of age [16]. Neuroblastoma is clinically significant, ranging from local spontaneous regression to positive progression. Despite current intensive multimodal therapy, children diagnosed with advanced neuroblastoma have poor prognosis, with an overall 5-year survival rate of about 40% [17]. In the past, clinical diagnosis of neuroblastoma was mainly based on surgical or puncture pathological results, but it had the disadvantages of poor tolerance and large trauma in children. Therefore, new auxiliary diagnosis methods are needed.

CT and MRI can effectively distinguish the location, shape, internal composition, and surrounding infiltration of the tumor, so as to achieve the purpose of accurate preoperative diagnosis.

The results of this study showed that the diagnostic accuracy of MRI was 96.15%, which was significantly higher than that of CT (78.85%, *P* < 0.05). ROC curve analysis results showed that the sensitivity, accuracy, and specificity indexes of MRI diagnosis were significantly better than those of CT diagnosis (*P* < 0.05), indicating that MRI diagnosis is of high value in the diagnosis of neuroblastoma of the central nervous system.

High expression of MYCN is in about 20% of neuroblastomas and is associated with tumor progression and poor prognosis. In recent years, it has been established that MYCN is an important regulator of several miRNAs in the formation of neuroblastoma. It has also been proved that cancer-suppressing miRNAs inhibit the expression of MYCN and the proliferation of neuroblastoma [18, 19]. This study showed that MYCN was highly expressed in the serum of neuroblastoma in children, suggesting that its overexpression may play an important role in activating the vitality of neuroblastoma tumor cells and may promote the growth, migration, and invasion ability of tumor cells. The results showed that the detection of serum MYCN mRNA level can effectively diagnose neuroblastoma. When CT and MRI combined with serum MYCN mRNA level detection, the diagnostic probability of neuroblastoma may be higher, and the sensitivity was significantly improved.

Tumor markers are markers that characterize malignant cells. In the process of tumor cell development, growth, metastasis, and invasion, tumor markers are substances that are synthesized, secreted, and released by tumor cell gene expression or abnormally produced and/or elevated by the body in response to tumor cells or secreted into body fluids. They are substances that reflect the occurrence and development of tumors and are conducive to diagnosis, differential diagnosis, and prognosis. LDH, NSE, CEA, and MYCN are all typical, among which serum NSE has stronger specificity for the diagnosis of NB. However, these biomarkers do not have a high early positive rate, which may be due to the low tumor load displayed in the early stage of the disease, resulting in unclear tumor marker characteristics. Through the combined diagnosis results, this study further suggested that CT and MRI combined with serum indicators could be used for comprehensive diagnosis in various aspects, thus improving the diagnostic efficiency and providing new ideas and clinical guidance for the early diagnosis of neuroblastoma.

## Conclusion

In conclusion, CT and MRI combined with serum indicators had high diagnostic value for pediatric neuroblastoma, and their sensitivity was significantly higher than that of the three separate diagnoses, which to a certain extent made up for the missed diagnosis caused by atypical imaging characteristics in ultrasonic diagnosis of neuroblastoma.

## Data Availability

The datasets used and/or analyzed during the present study are available from the corresponding author on reasonable request.
